# Supplementary material for: Spatial-extent inference for testing variance components in reliability and heritability studies
Source: Imaging Neurosci (Camb). 2024 Jan 9;2:imag-2-00058. doi: 10.1162/imag_a_00058 (PMC12224426; doi:10.1162/imag_a_00058)
Supplement: Supplementary Material [file imag_a_00058-supp.pdf]

# Supplementary materials for "Spatial-extent inference for testing variance components in reliability and heritability studies"

Ruyi Pan[1,2], Erin W. Dickie[2,3], Colin Hawco[2,3], Nancy Reid[1], Aristotle N. Voineskos[2,3], Jun Young Park[1,4*]

[1]*Department of Statistical Sciences, University of Toronto, Toronto, ON, Canada*
[2]*Centre for Addiction and Mental Health, Toronto, ON, Canada*
[3]*Department of Psychiatry, University of Toronto, Toronto, ON, Canada*
[4]*Department of Psychology, University of Toronto, Toronto, ON, Canada*

## A. Normal approximation of mixture chi-square distribution

Combing A.1 and A.2, the null distribution of $\sum_{v^* \in N_r(v)} U(v^*)$ is approximated by the normal distribution when $N$ is large. This implies that $T_r(v)$ (the standardized $\sum_{v^* \in N_r(v)} U(v^*)$) follows the standard normal distribution approximately under $H_0$.

### A.1 Proof that $\sum_{v^* \in N_r(v)} U(v^*)$ follows mixture chi-square under $H_0$

Based on our model specification, we define $\mathbf{y} = (y_1(1), y_2(1), \ldots, y_N(1), \ldots, y_1(V), y_2(V), \ldots, y_N(V))'$, which leads to

$$\mathbf{y} \sim \mathcal{MVN}((\mathbf{I}_V \otimes \mathbf{X})\boldsymbol{\beta}, \boldsymbol{\Sigma} \otimes \mathbf{I}_N)$$

where $\boldsymbol{\beta} = (\boldsymbol{\beta}(1)', \ldots, \boldsymbol{\beta}(V)')'$ and $\otimes$ denotes for the Kronecker product. Because OLS is used to estimate $\boldsymbol{\beta}(v)$, we have

$$\hat{\boldsymbol{\epsilon}} = \mathbf{y} - (\mathbf{I}_V \otimes \mathbf{X})\hat{\boldsymbol{\beta}} = \underbrace{[\mathbf{I}_{NV} - (\mathbf{I}_V \otimes \mathbf{X}(\mathbf{X}'\mathbf{X})^{-1}\mathbf{X}')]}_{\mathbf{P}}\mathbf{y}.$$

After estimating $\hat{\sigma}^2, \hat{\tau}^2$ and $\hat{\phi}$ by covariance regression analysis, we get $\hat{\boldsymbol{\Sigma}}^{-1} \otimes \mathbf{I}_N$ where $\hat{\boldsymbol{\Sigma}} = \hat{\sigma}^2\boldsymbol{\Phi}(\hat{\phi}, \mathcal{D}) + \hat{\tau}^2\mathbf{I}_V$. Then, we can get the $\hat{\mathbf{b}}$ by estimated conditional expectation $\hat{E}(\mathbf{b}|\mathbf{y})$ from the joint distribution of $(\mathbf{b}, \mathbf{y})$:

$$\begin{pmatrix}\mathbf{b}\\ \mathbf{y}\end{pmatrix} \sim \mathcal{MVN}\left(\begin{pmatrix}\mathbf{0}_{NV}\\ (\mathbf{I}_V \otimes \mathbf{X})\boldsymbol{\beta}\end{pmatrix}, \begin{pmatrix}\mathbf{I}_{NV} & \sigma^2\boldsymbol{\Phi}(\phi, \mathcal{D}) \otimes \mathbf{I}_N\\ \sigma^2\boldsymbol{\Phi}(\phi, \mathcal{D}) \otimes \mathbf{I}_N & \boldsymbol{\Sigma} \otimes \mathbf{I}_N\end{pmatrix}\right),$$

*Corresponding author: `junjy.park@utoronto.ca`

which provides

$$E(\mathbf{b}|\mathbf{y}) = \left(\sigma^2\mathbf{\Phi}(\phi,\mathcal{D})\otimes\mathbf{I}_N\right)\left(\mathbf{\Sigma}^{-1}\otimes\mathbf{I}_N\right)\left(\mathbf{y}-(\mathbf{I}_V\otimes\mathbf{X})\boldsymbol{\beta}\right).$$

Plugging in the estimated spatial parameters, we obtain

$$\begin{aligned}
\mathbf{y}-(\mathbf{I}_V\otimes\mathbf{X})\hat{\boldsymbol{\beta}}-\hat{\mathbf{b}} &= \mathbf{y}-(\mathbf{I}_V\otimes\mathbf{X})\hat{\boldsymbol{\beta}}-\left(\hat{\sigma}^2\mathbf{\Phi}(\hat{\phi},\mathcal{D})\otimes\mathbf{I}_N\right)(\hat{\mathbf{\Sigma}}^{-1}\otimes\mathbf{I}_N)\left(\mathbf{y}-(\mathbf{I}_V\otimes\mathbf{X})\hat{\boldsymbol{\beta}}\right)\\
&= \left[\mathbf{I}_{NV}-\left(\hat{\sigma}^2\mathbf{\Phi}(\hat{\phi},\mathcal{D})\otimes\mathbf{I}_N\right)(\hat{\mathbf{\Sigma}}^{-1}\otimes\mathbf{I}_N)\right]\hat{\boldsymbol{\epsilon}}\\
&= \left[\left(\hat{\mathbf{\Sigma}}\hat{\mathbf{\Sigma}}^{-1}\right)\otimes\mathbf{I}_N-\left(\hat{\sigma}^2\mathbf{\Phi}(\hat{\phi},\mathcal{D})\otimes\mathbf{I}_N\right)(\hat{\mathbf{\Sigma}}^{-1}\otimes\mathbf{I}_N)\right]\hat{\boldsymbol{\epsilon}}\\
&= \left[\left(\hat{\mathbf{\Sigma}}\hat{\mathbf{\Sigma}}^{-1}\right)\otimes\mathbf{I}_N-\left(\hat{\sigma}^2\mathbf{\Phi}(\hat{\phi},\mathcal{D})\hat{\mathbf{\Sigma}}^{-1}\right)\otimes\mathbf{I}_N)\right]\hat{\boldsymbol{\epsilon}}\\
&= \left[\left((\hat{\mathbf{\Sigma}}-\hat{\sigma}^2\mathbf{\Phi}(\hat{\phi},\mathcal{D}))\hat{\mathbf{\Sigma}}^{-1}\right)\otimes\mathbf{I}_N\right]\hat{\boldsymbol{\epsilon}}\\
&= \left[\left(\hat{\tau}^2\mathbf{I}_V\hat{\mathbf{\Sigma}}^{-1}\right)\otimes\mathbf{I}_N\right]\hat{\boldsymbol{\epsilon}}\\
&= \hat{\tau}^2(\hat{\mathbf{\Sigma}}^{-1}\otimes\mathbf{I}_N)\hat{\boldsymbol{\epsilon}}.
\end{aligned}$$

To get corresponding test statistics for each vertex $v$, we define $\mathbf{S}_v:\mathbb{R}^{NV}\to\mathbb{R}^N$ to identify the subset of all images' element for vertice $v$ (e.g., $\mathbf{S}_{\{1\}}\hat{\boldsymbol{\epsilon}}=\hat{\boldsymbol{\epsilon}}(1)$). Then, the test statistic becomes

$$\begin{aligned}
U(v) &= \hat{\boldsymbol{\epsilon}}'(\hat{\mathbf{\Sigma}}^{-1}\otimes\mathbf{I}_N)\hat{\tau}^2\mathbf{S}'_{\{v\}}\mathbf{K}\mathbf{S}_{\{v\}}\hat{\tau}^2(\hat{\mathbf{\Sigma}}^{-1}\otimes\mathbf{I}_N)\hat{\boldsymbol{\epsilon}}\\
&= \mathbf{y}'\mathbf{P}\underbrace{\left[(\hat{\mathbf{\Sigma}}^{-1}\otimes\mathbf{I}_N)'\hat{\tau}^2\mathbf{S}'_{\{v\}}\mathbf{K}\mathbf{S}_{\{v\}}\hat{\tau}^2(\hat{\mathbf{\Sigma}}^{-1}\otimes\mathbf{I}_N)\right]}_{\mathbf{A}}\mathbf{P}\mathbf{y}.
\end{aligned}$$

Similarly, define $\mathbf{S}_{N_r(v)}:\mathbb{R}^{NV}\to\mathbb{R}^{N\cdot|N_r(v)|}$ to identify the subset of all subjects' elements for vertices in $N_r(v)$ (e.g., $\mathbf{S}_{\{1,2\}}\hat{\boldsymbol{\epsilon}}=(\hat{\boldsymbol{\epsilon}}(1)',\hat{\boldsymbol{\epsilon}}(2)')'$. Then, for cluster-enhanced test statistic, we have

$$\sum_{v^*\in N_r(v)}U(v^*) = \mathbf{y}'\mathbf{P}\underbrace{\left[(\hat{\mathbf{\Sigma}}^{-1}\otimes\mathbf{I}_N)'\hat{\tau}^2\mathbf{S}'_{N_r(v)}(\mathbf{I}_{|N_r(v)|}\otimes\mathbf{K})\mathbf{S}_{N_r(v)}\hat{\tau}^2(\hat{\mathbf{\Sigma}}^{-1}\otimes\mathbf{I}_N)\right]}_{\mathbf{A}}\mathbf{P}\mathbf{y}. \tag{1}$$

Since $\mathbf{P}\mathbf{y}$ follows multivariate normal with zero mean vector and the covariance matrix $\mathbf{P}(\mathbf{\Sigma}\otimes\mathbf{I}_N)\mathbf{P}$, $\mathbf{y}'\mathbf{P}\mathbf{A}\mathbf{P}\mathbf{y}$ follows a mixture chi-square distribution with weights being the eigenvalues of $\mathbf{Q}\mathbf{A}\mathbf{Q}'$ where $\mathbf{Q}$ corresponds to the Cholesky decomposition of $\mathbf{Q}\mathbf{Q}'=\mathbf{P}(\hat{\mathbf{\Sigma}}\otimes\mathbf{I}_N)\mathbf{P}$ (Duchesne & De Micheaux, 2010).

## A.2 Proof that the mixture chi-square distribution converges in distribution to a normal distribution

We use the Lyapunov Central Limit Theorem to prove the mixture chi-square distribution converges in distribution to a normal distribution as the number of components in the mixture chi-square goes to infinity. Assume $\{Y_1,\ldots,Y_k\}$ is a sequence of independent random variables, each with finite expected value $\mu_i$ and variance $\sigma_i^2$, and $s_k^2=\sum_{i=1}^k\sigma_i^2$. The Lyapunov Central Limit Theorem

states that if the following condition is satisfied for the sequence:

$$\lim_{k\to\infty} \frac{1}{s_k^{2+\delta}} \sum_{i=1}^{k} \mathrm{E}[\mid Y_i - \mu_i \mid^{2+\delta}] = 0 \quad \text{for some } \delta > 0,$$

then $\sum_{i=1}^{k}(Y_i - \mu_i)/s_k$ will converge weakly to the standard normal distribution.

Suppose $X_1, \ldots, X_k \overset{i.i.d}{\sim} \chi_1^2$ are the components of the mixture chi-square for our test statistic (1) with their corresponding weights $\lambda_1, \ldots, \lambda_k$ which are the eigenvalues of $\mathbf{QAQ}^{'}$. Then it is sufficient to prove that the sequence of $\lambda_i X_i$ satisfies the Lyapunov's condition. Because the fourth central moment of $\chi_1^2$ is 48, we can get the fourth central moment of $\lambda_i X_i$: $\mathrm{E}[(\lambda_i X_i - \lambda_i \mu_i)^4] = \lambda_i^4 \mathrm{E}[(X_i - \mu_i)^4] = 48\lambda_i^4$. Then, choosing $\delta = 2$, we get

$$\sum_{i=1}^{k} \mathrm{E}[(\lambda_i X_i - \lambda_i \mu_i)^4] = 48 \sum_{i=1}^{k} \lambda_i^4.$$

Because $\mathrm{Var}(\lambda_i X_i) = \lambda_i^2 \mathrm{Var}(X_i) = 2\lambda_i^2$, we have

$$s_k^4 = \left( \sum_{i=1}^{k} \mathrm{Var}(\lambda_i X_i) \right)^2 = 4 \left( \sum_{i=1}^{k} \lambda_i^2 \right)^2 .$$

The result above leads to

$$\frac{1}{s_k^4} \sum_{i=1}^{n} \mathrm{E}[(\lambda_i X_i - \lambda_i \mu_i)^4] = \frac{48 \sum_{i=1}^{k} \lambda_i^4}{4 \left( \sum_{i=1}^{k} \lambda_i^2 \right)^2} \leq \frac{12}{k} \left( \frac{\lambda_{\max}}{\lambda_{\min}} \right)^4 ,$$

where $\lambda_{\max}$ and $\lambda_{\min}$ are the largest and smallest eigenvalues, respectively. As $k$ goes to infinity,

$$\lim_{k\to\infty} \frac{1}{s_k^4} \sum_{i=1}^{n} \mathrm{E}[(\lambda_i X_i - \lambda_i \mu_i)^4] \leq \lim_{k\to\infty} \frac{12}{k} \left( \frac{\lambda_{\max}}{\lambda_{\min}} \right)^4 = 0,$$

provided that $\lambda_{\max}/\lambda_{\min}$ is bounded. Therefore, the Lyapunov's condition is satisfied and $\sum_{i=1}^{k}(\lambda_i X_i - \lambda_i \mu_i)/s_k$ converges in distribution to a standard normal random variable which is equivalent to $\sum_{i=1}^{k} \lambda_i X_i$ converges weakly to a normal distribution.

**B. Proof that $T_r(v)$ is constructed by assuming $\theta^2(v^*)$s in the neighborhood $N_r(v)$ are the same**

Let $v_1, \dots, v_{|N_r(v)|}$ be indices of vertices in $N_r(v)$. From our model assumptions, the model within $N_r(v)$ is

$$\begin{pmatrix} \mathbf{y}(v_1) \\ \mathbf{y}(v_2) \\ \vdots \\ \mathbf{y}(v_{|N_r(v)|}) \end{pmatrix} = \begin{pmatrix} \mathbf{X}\boldsymbol{\beta}(v_1) \\ \mathbf{X}\boldsymbol{\beta}(v_2) \\ \vdots \\ \mathbf{X}\boldsymbol{\beta}(v_{|N_r(v)|}) \end{pmatrix} + \begin{pmatrix} \mathbf{r}(v_1) \\ \mathbf{r}(v_2) \\ \vdots \\ \mathbf{r}(v_{|N_r(v)|}) \end{pmatrix} + \begin{pmatrix} \boldsymbol{\epsilon}(v_1) \\ \boldsymbol{\epsilon}(v_2) \\ \vdots \\ \boldsymbol{\epsilon}(v_{|N_r(v)|}). \end{pmatrix}$$

Assuming that $\theta^2(v^*)$s in $N_r(v)$ have the same value $\theta^2$, then

$$\begin{pmatrix} \mathbf{r}(v_1) \\ \mathbf{r}(v_2) \\ \vdots \\ \mathbf{r}(v_{|N_r(v)|}) \end{pmatrix} \sim \mathcal{MVN}\left(\mathbf{0}_{N\cdot|N_r(v)|}, \theta^2 \times \mathbf{K} \otimes \mathbf{I}_{|N_r(v)|}\right).$$

Since the $\hat{\boldsymbol{\beta}}(v)$s and $\hat{\mathbf{b}}(v)$ are obtained from the null model, they keep the same estimated value as we described in section 2.3. Within the $N_r(v)$, the score-based variance component test statistic for $\theta^2$ becomes

$$\begin{pmatrix} \mathbf{y}(v_1) - \mathbf{X}\hat{\boldsymbol{\beta}}(v_1) - \hat{\mathbf{b}}(v_1) \\ \mathbf{y}(v_2) - \mathbf{X}\hat{\boldsymbol{\beta}}(v_2) - \hat{\mathbf{b}}(v_2) \\ \vdots \\ \mathbf{y}(v_{|N_r(v)|}) - \mathbf{X}\hat{\boldsymbol{\beta}}(v_{|N_r(v)|}) - \hat{\mathbf{b}}(v_{|N_r(v)|}) \end{pmatrix}' \mathbf{K} \otimes \mathbf{I}_{|N_r(v)|} \begin{pmatrix} \mathbf{y}(v_1) - \mathbf{X}\hat{\boldsymbol{\beta}}(v_1) - \hat{\mathbf{b}}(v_1) \\ \mathbf{y}(v_2) - \mathbf{X}\hat{\boldsymbol{\beta}}(v_2) - \hat{\mathbf{b}}(v_2) \\ \vdots \\ \mathbf{y}(v_{|N_r(v)|}) - \mathbf{X}\hat{\boldsymbol{\beta}}(v_{|N_r(v)|}) - \hat{\mathbf{b}}(v_{|N_r(v)|}) \end{pmatrix}.$$

Note that, this matrix multiplication is summarized by

$$\sum_{v^* \in N_r(v)} (\mathbf{y}(v^*) - \mathbf{X}\hat{\boldsymbol{\beta}}(v^*) - \hat{\mathbf{b}}(v_i))'\mathbf{K}(\mathbf{y}(v^*) - \mathbf{X}\hat{\boldsymbol{\beta}}(v^*) - \hat{\mathbf{b}}(v^*))$$

which is equal to $\sum_{v \in N_r(v)} U(v^*)$. This result suggests that $T_r(v)$ is constructed by assuming the $\theta^2(v)s$ are same in the neighborhood $N_r(v)$ as we stated in Section 2.3.

## C. Supplementary figures

Figures S1 and S2 show binarized maps of statistical significance when CLEAN-V, CLEAN-V without spatial correlation, CLEAN-V without cluster enhancement, and massive univariate analysis are applied to language, social cognition, relational processing, and gambling tasks.

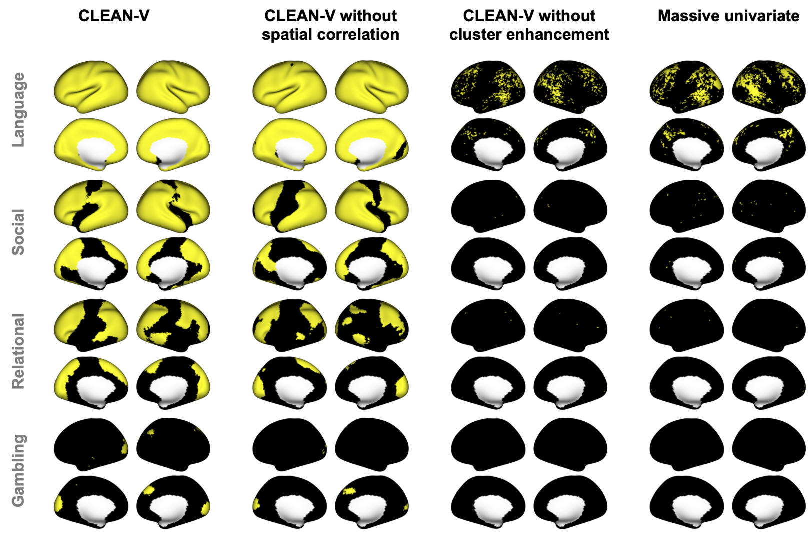


**Figure S1**: Test-retest reliability localization results from the four methods for the other four tasks.

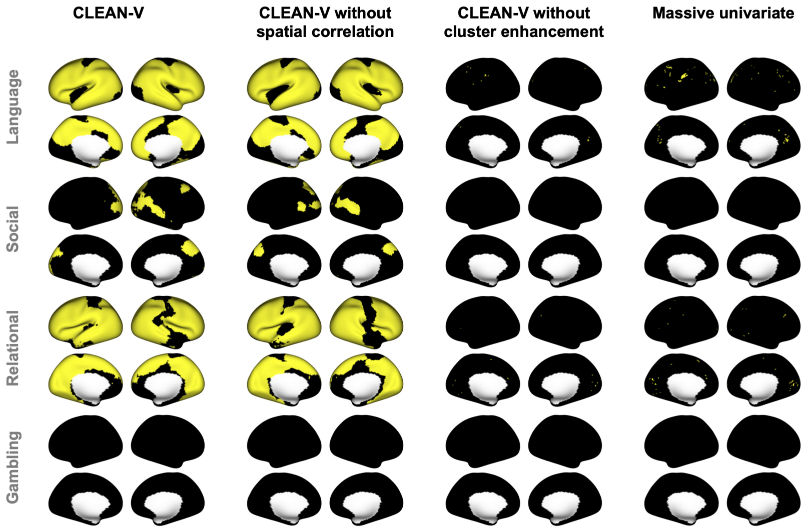


**Figure S2**: Narrow-sense heritability localization results from the four methods for the four other tasks.

## D. Evaluation of FWER of the score test by Ganjgahi et al. (2015)

We used simulation setup in Section 3.4.2 to evaluate the family-wise error rate of the score test by Ganjgahi et al. (2015). We used 'Null model residual permutation (P2)' to obtain the null distribution and the max$T$ approach to control FWER. Figure S3 shows the Family-wise error rates of CLEAN-V and its competitors when $\alpha = 0.05$ (5%) was used to set a threshold for each simulated dataset. Clearly, CLEAN-V and MEGHA controlled FWER accurately. However, Ganjgahi's score test had inflated FWER (around 15%) regardless of the degrees of smoothing applied. The codes we used to implement the method is available at `https://github.com/RuyiPan/Code4Review-CLEAN-V`.

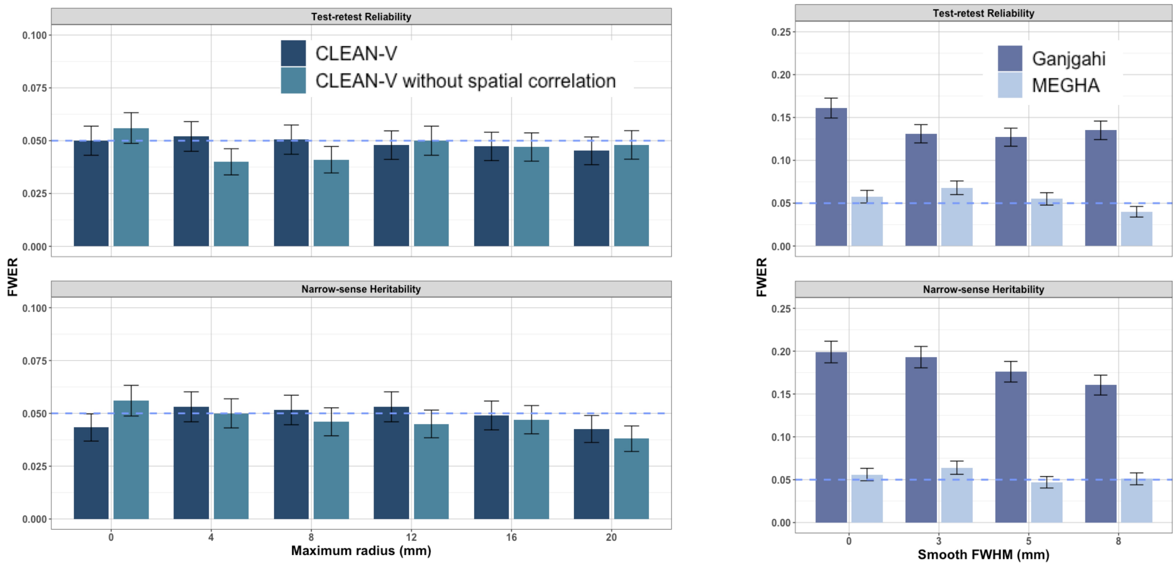


**Figure S3**: On the left side: Family-wise error rates of CLEAN-V and its competitors under different maximum radii $r_{\text{max}}$s from the data-driven simulation studies under null data of Test-retest reliability or Narrow-sense heritability study (no true test-retest images/twin images). The x-axis label refers to the maximum radius for CLEAN-V-type clusterwise enhancement. On the right side: Family-wise error rates of Ganjgahi's score test with smoothed images. The x-axis label refers to the surface smoothing parameter FWHM.

## References


Duchesne, P., & De Micheaux, P. L. (2010). Computing the distribution of quadratic forms: Further comparisons between the liu–tang–zhang approximation and exact methods. *Computational Statistics & Data Analysis*, *54*(4), 858–862. doi: https://doi.org/10.1016/j.csda.2009.11.025

Ganjgahi, H., Winkler, A. M., Glahn, D. C., Blangero, J., Kochunov, P., & Nichols, T. E. (2015). Fast and powerful heritability inference for family-based neuroimaging studies. *NeuroImage*, *115*, 256–268. doi: https://doi.org/10.1016/j.neuroimage.2015.03.005
